# An Agomir of miR-144-3p Accelerates Plaque Formation through Impairing Reverse Cholesterol Transport and Promoting Pro-Inflammatory Cytokine Production

**DOI:** 10.1371/journal.pone.0094997

**Published:** 2014-04-14

**Authors:** Yan-Wei Hu, Ya-Rong Hu, Jia-Yi Zhao, Shu-Fen Li, Xin Ma, Shao-Guo Wu, Jing-Bo Lu, Yu-Rong Qiu, Yan-Hua Sha, Yan-Chao Wang, Ji-Juan Gao, Lei Zheng, Qian Wang

**Affiliations:** 1 Laboratory Medicine Center, Nanfang Hospital, Southern Medical University, Guangzhou, Guangdong, China; 2 Department of Anesthesiology, Nanfang Hospital, Southern Medical University, Guangzhou, Guangdong, China; 3 Department of Vascular Surgery, Nanfang Hospital, Southern Medical University, Guangzhou, Guangdong, China; Harvard Medical School, United States of America

## Abstract

**Aims:**

ATP-binding cassette transporter A1 (ABCA1) mediates the efflux of cholesterol and phospholipids to lipid-poor apolipoproteins, which then form nascent HDL, a key step in the mechanism of reverse cholesterol transport (RCT). While a series of microRNAs (miRNAs) have been identified as potent post-transcriptional regulators of lipid metabolism, their effects on ABCA1 function and associated mechanisms remain unclear.

**Methods and Results:**

ABCA1 was identified as a potential target of miR-144-3p, based on the results of bioinformatic analysis and the luciferase reporter assay, and downregulated after transfection of cells with miR-144-3p mimics, as observed with real-time PCR and western blot. Moreover, miR-144-3p mimics (agomir) enhanced the expression of inflammatory factors, including IL-1β, IL-6 and TNF-α, *in vivo* and *in vitro*, inhibited cholesterol efflux in THP-1 macrophage-derived foam cells, decreased HDL-C circulation and impaired RCT *in vivo*, resulting in accelerated pathological progression of atherosclerosis in apoE^−/−^ mice. Clinical studies additionally revealed a positive correlation of circulating miR-144-3p with serum CK, CK-MB, LDH and AST in subjects with AMI.

**Conclusions:**

Our findings clearly indicate that miR-144-3p is essential for the regulation of cholesterol homeostasis and inflammatory reactions, supporting its utility as a potential therapeutic target of atherosclerosis and a promising diagnostic biomarker of AMI.

## Introduction

Atherosclerotic cardiovascular disease remains the leading cause of morbidity and mortality worldwide. Epidemiological studies have revealed that blood lipid abnormality is a key risk factor. High-density lipoprotein cholesterol (HDL-C) level is inversely correlated with rates of coronary events, and therefore considered cardioprotective [1]–[2]. Recent advances have established a fundamental role for inflammation in mediating all stages of this disease from initiation through progression of atherosclerosis [3]. These findings reveal new insights into the significant associations between risk factors and the mechanisms of atherogenesis.

Several ATP-binding cassette (ABC) transporters, such as ABCA1 and ABCG5, have been shown to play important roles in the regulation of cell cholesterol homeostasis [4]. In extrahepatic cells and tissues, ABCA1 mediates the efflux of cholesterol and phospholipids to lipid-poor apolipoproteins, which subsequently form nascent HDL, a key step in the mechanism of reverse cholesterol transport (RCT). Deficiency in ABCA1 is reported to lead to a dramatic reduction in plasma HDL levels. Mutations in the ABCA1 gene result in Tangier disease, a rare inherited disorder characterized by severe reduction in the amount of HDL and increased risk of atherosclerosis [5], [6].

In addition to classic transcriptional regulation of cholesterol metabolism, members of a class of microRNAs (miRNAs) have been identified as potent post-transcriptional regulators of lipid metabolism. Recently, miRNA-33a and b (miR-33a/b) were identified as key regulators of metabolic programs, including cholesterol and fatty acid homeostasis. These intronic miRNAs are embedded in the genes for sterol response element-binding proteins, SREBF2 and SREBF1, which code for transcription factors that modulate cholesterol and fatty acid synthesis [7], [8]. In addition to miR-33a/b, miR-122, miR-370, miR-335, miR-378/378*, miR-27 and miR-125a-5p have been implicated in regulating cholesterol homeostasis, fatty acid metabolism and lipogenesis [9].

Data from the current study showed that miR-144-3p mimics (agomir) suppress the expression of ABCA1 and enhance inflammatory factors, both *in vitro* and *in vivo*, inhibit cholesterol efflux in THP-1 macrophage-derived foam cells and impair RCT in apoE^−/−^ mice fed a high-fat diet (HFD), leading to accelerated pathological progression of atherosclerosis in experimental mice. Clinical studies additionally revealed a significant association between circulating miR-144-3p and acute myocardial infarction (AMI). These findings collectively suggest that miR-144-3p may be effectively used as a potential therapeutic target of atherosclerosis and promising diagnostic biomarker of AMI.

## Materials and Methods

Our study was conformed with the Declaration of Helsinki and approved by the Institutional Review Board of Nanfang Hospital, Southern Medical University (Guangzhou, China) and written informed consent was obtained from all subjects.

### Animals

This investigation conforms with the Guide for the Care and Use of Laboratory Animals published by the US National Institutes of Health (NIH Publication No. 85–23, revised 1996), and was approved by the Animal Experimental Committee at Nanfang Hospital. Male six-week-old apoE^−/−^ mice with a C57BL/6 background were purchased from Vital River Laboratory Animal Technology Co, Ltd (Beijing, China). Mice were randomized into two groups (Control and miR-144-3p agomir, n = 20/group) and injected via the tail vein with either a scrambled miRNA agomir (GuangZhou RiboBio. Co.) or miRNA analog (agomir) of mir-144-3p (GuangZhou RiboBio. Co.) at a dose of 20 mg/kg/day in 0.2 ml saline twice a week. Five animals were housed per cage at 25°C under a 12-h light/dark cycle. The mice were fed a high-fat diet (HFD) for a period of 12 weeks. The diet is a commercially prepared mouse food supplemented with 21% (wt/wt) butterfat, 0.15% (wt/wt) cholesterol, and 19.5% (wt/wt) casein (Beijing Keao Xieli Feed Co.LTD., Beijing). At week 12, mice were anesthetized, and 1 mL of blood collected via cardiac puncture before sacrifice via cervical dislocation. Tissues were collected for further analysis.

### Cell Culture

Human monocytic THP-1 cells were obtained from American Type Culture Collection (ATCC, Manassas, VA, USA). THP-1 cells were maintained in Roswell Park Memorial Institute (RPMI) 1640 medium containing 10% fetal calf serum (FCS) and differentiated for 72 h with 100 nM phorbol 12-myristate 13-acetate (PMA). Macrophages were transformed into foam cells by incubation in the presence or absence of 50 µg/mL ox-LDL in serum-free RPMI 1640 containing 0.3% BSA for 48 h. All cells were incubated at 37°C in an atmosphere of 5% CO_2_, seeded in 6- or 12-well plates or 60-mm dishes, and grown to 60–80% confluence before use.

### Isolation and Culture of Primary Human Macrophages

Peripheral blood mononuclear cells were prepared from forearm venous blood of healthy volunteers using Biocoll density gradient centrifugation, as described previously [10]. Monocytes (CD14+) were selected using paramagnetic beads according to the manufacturer's instructions (Miltenyi Biotech, Bergisch Gladbach, Germany), cultured in RPMI 1640 containing 10% homologous or AB serum on poly-L-lysine-coated 6-well plates at a density of 2 million cells per well, and incubated at 37°C under 5% CO_2_. All cell culture experiments were performed after 8 days of differentiation. For cells in different experimental settings, supplemented RPMI 1640 was replaced with serum-free RPMI 1640. Macrophage differentiation of primary human monocytes was monitored morphologically via light microscopy and fluorescence-activated cell sorting (FACS) analysis using a monoclonal antibody specific for CD163.

### Transfection of miRNA Mimics

THP-1 macrophages were transfected with 50 nM miRNA mimic (hsa-miR-144-3p, UACAGUAUAGAUGAUGUACU) using Lipofectamine 2000 transfection reagent for 48 h, according to the manufacturer's instructions. All experimental control samples were treated with equal concentrations of a non-targeting control mimic sequence (negative control, UUUGUACUACACAAAAGUACUG).

### Luciferase Assay

Human ABCA1 cDNA containing putative (wild-type) and mutated target sites for hsa-miR-144-3p was chemically synthesized, and inserted into pMIR-REPORT vector (Ambion, Austin, TX, USA). The pMIR-REPORT beta-galactosidase control vector (Ambion) was used as a reference. For the luciferase assay, 293T cells (human embryonic kidney) were co-transfected with wild-type (pMIR-ABCA1-wt) or mutant (pMIR-ABCA1-mt) reporter vectors and hsa-miR-144-3p mimics, using Lipofectamine 2000 transfection reagent. Luciferase activity was measured at 48 h post-transfection using a dual-luciferase assay kit (Promega, Madison, WI, USA).

### RNA Isolation and Real-time Quantitative PCR

Total RNA from tissues or cultured cells was extracted using TRIzol reagent (Invitrogen, Carlsbad, CA, USA), according to the manufacturer's instructions. Total RNA from 200 µL serum was isolated using the miRVana PARIS kit (Ambion, Austin, TX, USA). mir-144-3p was detected with the All-in-One miRNA qPCR Kit (GeneCopoeia, Rockville, MD, USA) in a 20 µL reaction volume, using the manufacturer's protocol. Real-time quantitative PCR (qRT-PCR) was performed on the ABI 7500 Fast system (Applied Biosystems, Foster City, CA, USA). U6 RNA expression was used as the endogenous control for analysis of miR-144-3p from cells and tissues. For serum miR-144-3p analysis, isolation and assay were performed using a series of concentrations of miR-144-3p (synthesized by IDT, Coralville, IA, USA) to generate a standard curve. The absolute amount of mir-144-3p was calculated with software based on sample qRT-PCR numbers and the standard curve, and expressed as pmol/L. mRNA levels were evaluated with qRT-PCR using an ABI 7500 Fast Real Time PCR system with SYBR Green detection chemistry (TaKaRa Bio, Inc., Shiga, Japan). Expression of glyceraldehyde-3-phosphate dehydrogenase was used as the internal control. Quantitative measurements were performed using the ΔΔCt method. All samples were measured in triplicate, and mean values considered for comparative analysis.

### Western Blot Analyses

Cells and tissues were harvested and protein extracts prepared using established methods. Extracts were separated using 10% sodium dodecyl sulfate polyacrylamide gel electrophoresis and subjected to western blot using rabbit polyclonal anti-ABCA1 antibodies (Abcam Inc., Cambridge, MA, USA) and rabbit polyclonal anti-β-actin antibody (Santa Cruz Biotechnologies, Inc., Santa Cruz, CA, USA). Proteins were visualized using the chemiluminescence method (ECL Plus Western Blot Detection System; Amersham Biosciences, Foster City, CA, USA).

### Cytokine Assays and Measurement of Serum Biochemical Parameters

Human IL-1β, IL-6 and TNF-α contents in culture medium (R&D Systems, Minneapolis, MN, USA), serum concentrations of IL-1β, IL-6 and TNF-α (R&D Systems, Minneapolis, MN, USA), and levels of serum apolipoprotein A1 and apolipoprotein B (Cusabio Biotech Co., Ltd., Wuhan, China) were measured using ELISA according to the manufacturer's instructions. Plasma TC and TG levels were analyzed in an automated analyzer (Beckman AU5400). Lipoproteins were isolated by sequential ultracentrifugation from 60 µl of plasma at densities (d) of <1.006 g/mL (very-low-density lipoprotein), 1.006≤d≤1.063 g/mL (intermediate-density lipoprotein and low-density lipoprotein) and d>1.063 g/mL (high density lipoprotein) in a LE-80K ultracentrifuge (Beckman). Cholesterol in the lipoprotein fractions was determined enzymatically using a colorimetric method (Beckman AU5400).

### HPLC Analysis of Cellular Cholesterol Levels

High-performance liquid chromatography (HPLC) analysis was conducted, as described previously [11]. Sterol analyses were performed using a HPLC system (model 2790, controlled with Empower Pro software; Waters Corp., Milford, MA, USA). Absorbance at 216 nm was monitored, and data analyzed with TotalChrom software from PerkinElmer (Waltham, MA, USA).

### Cellular Cholesterol Efflux Experiments

THP-1 macrophages were labeled with 0.2 µCi/mL [^3^H] cholesterol and cholesterol-loaded using 50 µg/ml oxidized LDL. After 72 h, cells were washed with PBS and incubated overnight in RPMI 1640 containing 0.1% (w/v) BSA to allow equilibration of [^3^H] cholesterol in all cellular pools. Equilibrated [^3^H] cholesterol-labeled cells were washed with PBS and incubated in 2 mL efflux medium containing RPMI 1640 and 0.1% BSA with 25 µg/mL of human plasma apoAI or 100 µg/mL of human plasma HDL for 12 h. A 150 µL sample of efflux medium was obtained at the designated times and passed through a 0.45-µm filter to remove floating cells. Monolayers were washed twice with PBS, and cellular lipids extracted with isopropanol. Medium and cell-associated [^3^H] cholesterol were measured using liquid scintillation counting. Percent efflux was calculated using the following equation: [total media count/(total cellular count + total media count)]×100%.

### *In Vivo* RCT Assay

Bone marrow–derived macrophages were prepared from C57BL/6 mice, as described previously [12]. Bone marrow was isolated, and cells were plated overnight in DMEM supplemented with 10% FBS and 15% L-929 conditioned medium. Non-adherent cells were removed and cultured for an additional 6 days to allow for macrophage differentiation. For RCT assays, bone marrow-derived macrophages (BMDMs) were washed twice and incubated with 37.5 µg/mL of acetylated LDL (Ac-LDL) and 5 µCi/mL of ^3^H-cholesterol for 24 h [13]. Cells were resuspended in ice-cold DMEM, and an aliquot (3×10^6^ cells) injected subcutaneously into individually housed mice treated with either scrambled miRNA agomir or miRNA analog (agomir) of miR-144-3p for 12 weeks, as described above. Prior to injection, an aliquot of cells was quantified using liquid scintillation counting to measure baseline radioactivity. Blood was obtained via saphenous vein puncture at 6, 12, and 24 h after BMDM injection and cardiac puncture after 48 h at sacrifice. An aliquot of plasma was used for liquid scintillation counting immediately at each time-point. Feces were collected for 48 h after injection, homogenized in 50% NaOH overnight, and an aliquot used for liquid scintillation counting. At sacrifice, animals were perfused with PBS to remove the blood and then liver samples were collected and incubated with hexane/isopropanol (3∶2) for 48 h and dried overnight. Lipids were resolubilized in liquid scintillation fluid, and radioactivity counted. RCT to plasma, liver, and feces was calculated as a percentage of total radioactivity injected at baseline.

### En Face Plaque Area

After mice were sacrificed, aortas were excised immediately and fixed in 10% buffered formalin for quantification of the en face plaque areas. Briefly, after adventitial tissue was carefully removed, the aorta was opened longitudinally, stained with Oil Red O (Sigma), and pinned on a blue wax surface. En face images were obtained under a stereomicroscope (SZX12; Olympus, Tokyo, Japan) equipped with a digital camera (Dxm1200, Nikon, Tokyo, Japan) and analyzed using Adobe Photoshop version 7.0 and Scion Image software. The luminal surface area stained with Oil Red O was determined as a percentage [14].

### Quantification of Atherosclerosis in the Aortic Sinus

The upper portion of the heart and proximal aorta were obtained, embedded in Optimal Cutting Temperature (OCT) compound (Fisher, Tustin, CA), and stored at −70°C. Serial 10-µm thick cryosections of aorta, beginning at the aortic root, were collected for a distance of 400 µm. Sections were stained with Oil Red O. Aortic root atherosclerosis was assessed as the average of three sections, each separated by 100 µm, beginning at the site of appearance of the coronary artery and valve leaflets. The Oil Red O-positive areas in digitized color images of stained aortic root sections were quantified using Image-Pro Plus image analysis software (Media Cybernetics, Rockville, MD, USA), and data expressed as a percentage of the total section area.

### Blood Samples

AMI was diagnosed, based on a combination of several criteria: 1) ischemic symptoms, 2) increased cardiac cTnI level, 3) creatine kinase-MB (CK-MB), 4) pathological Q wave, and 5) ST-segment elevation or depression [15]. In total, 25 healthy volunteers (with normal electrocardiogram and no history of cardiovascular disease) were enrolled in this study. Blood samples of patients with AMI were obtained at 4 h (±30 min), 8 h (±30 min), 12 h (±30 min), 24 h (±30 min), 48 h (±30 min), 72 h (±30 min) and 1 week (±60 min) after the onset of symptoms. Plasma was isolated via centrifugation and maintained at −80°C until RNA extraction.

### Statistical Analysis

Data are expressed as mean values ± standard deviations (SD). Results were analyzed with one-way ANOVA analysis of variance followed by the Student-Newman-Keuls (SNK) test and the Student's *t*-test using SPSS v13.0 statistical software (SPSS, Inc., Chicago, IL, USA). A two-tailed probability (*p*) value <0.05 was considered statistically significant.

## Results

### 1. Effects of miR-144-3p on ABCA1 expression, cholesterol homeostasis, and inflammation in THP-1 macrophages

**Figure 1:**
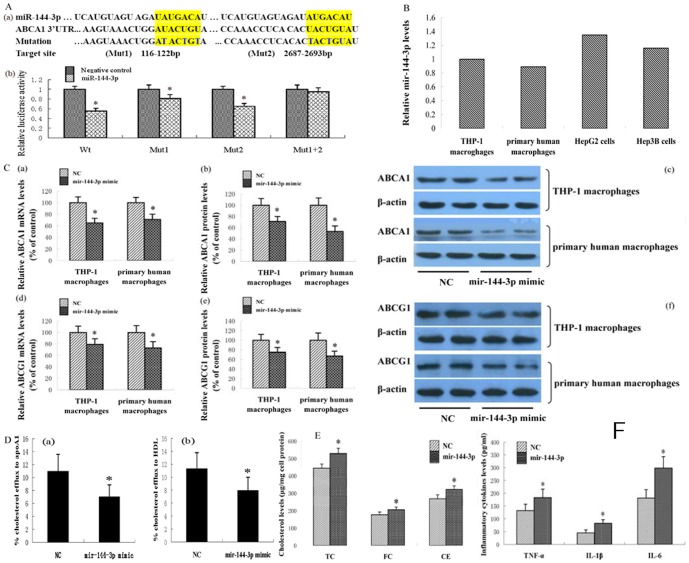
Effects of miR-144-3p on ABCA1 expression, cholesterol homeostasis and inflammation. (A) hsa-miR-144-3p directly targeted the 3′-untranslated region (3′UTR) of ABCA1. (a) Sequence alignment of the human hsa-miR-144-3p mature sequence with the binding sites of human ABCA1 3′UTR. (b) Changes in luciferase activity induced by the hsa-miR-144-3p mimic in binding to ABCA1 3′-UTR (n = 3; *p<0.01 vs. 293T cells transfected with negative control miRNAs). (B) miR-144-3p expression levels in various cells were analyzed by qRT-PCR. (C) THP-1 macrophages and primary macrophages were exposed to 50 µg/mL of oxidized LDL for 48 h and then treated with 50 nM miRNA mimics, as indicated, for 48 h. ABCA1 mRNA and protein levels were measured using qRT-PCR and western blot analysis, respectively. (D) THP-1 cells were differentiated for 72 h with 100 nM PMA and then macrophages were treated with 50 nM miRNA mimics for 48 h. Then, the cells were labeled with 0.2 µCi/mL [^3^H] cholesterol and cholesterol-loaded using 50 µg/ml oxidized LDL. The percentage of cholesterol efflux to (a) apoAI and (b) HDL was analyzed with the liquid scintillation counting assay. (E) THP-1 cells were differentiated for 72 h with 100 nM PMA and then macrophages were transformed into foam cells by incubation in the presence of 50 µg/mL of oxidized LDL for 48 h. THP-1 macrophage-derived foam cells were treated with 50 nM miRNA mimics for 48 h, as indicated, and the cholesterol content measured using HPLC. (F) THP-1 cells were differentiated for 72 h with 100 nM PMA and then macrophages were transformed into foam cells by incubation in the presence of 50 µg/mL of oxidized LDL for 48 h. THP-1 macrophage-derived foam cells were treated with 50 nM miRNA mimics, as indicated, for 48 h, and inflammatory cytokines in the medium measured with ELISA. All results are expressed as mean values ± S.D. of three independent experiments, each performed in triplicate. *p<0.05 vs. control group.

ABCA1 is known to play a key role in cholesterol homeostasis and anti-atherosclerosis. Here, we identified a putative miRNA, designated miR-144-3p, which targets human ABCA1, utilizing a combination of TargetScan, miRanda, RNA22, miRDB, miRGen, PITA, EMBL-EBI, starBase, PicTar, RNAhybrid and miRBase. The sequence and seed pairing of this miRNA are conserved across multiple species. To establish the effect of miR-144-3p on the 3′UTR of human ABCA1, the dual luciferase assay was performed. As shown in Fig. 1A, human ABCA1 mRNA contains two putative complementary sequences to miR-144-3p. Upon co-transfection of 293T cells with wild-type (pMIR-ABCA1-wt) reporter vectors and hsa-miR-144-3p using Lipofectamine 2000 transfection reagent, luciferase activity was significantly decreased. Mutation of binding site 1 or 2 markedly suppressed the effect of hsa-miR-144-3p, while mutation of both binding sites completely reversed the effect of hsa-miR-144-3p.

Macrophages play a central role in atherosclerosis, and ABCA1 is critical for cellular cholesterol efflux. Accordingly, we transfected THP-1 macrophages and human primary macrophages with miR-144-3p mimics or negative control to establish the effect of miR-144-3p on ABCA1 expression. Our results showed that miR-144-3p mimics significantly suppress the ABCA1 mRNA and protein levels in the two cell models (Fig. 1B). Cells transport excess cholesterol across, in part, through the ABCA1 pathway to prevent toxicity resulting from cholesterol overload [16]. To investigate the impact of miR-144-3p on cholesterol homeostasis, cultured THP-1 macrophages were transfected with miR-144-3p mimics. Cholesterol efflux to both apoAI and HDL was markedly decreased, compared to the control group (Fig. 1C). Additionally, total cholesterol, free cholesterol and cholesterol ester levels in THP-1 macrophage-derived foam cells were significantly increased as a result of treatment with miR-144-3p mimics (Fig. 1D).

Atherosclerosis is a complex inflammatory disease, with macrophage foam cells being the major cell type involved in inflammation [17]–[18]. ABCA1 has been shown to have anti-inflammatory activity independent of its role in RCT. Therefore, we investigated the effects of miR-144-3p mimics on inflammatory factor expression in THP-1 macrophage-derived foam cells. Notably, following treatment with miR-144-3p mimics, secretion of inflammatory cytokines, including TNF-α, IL-1β and IL-6, into culture medium was dramatically increased (Fig. 1E).

### 2. Effects of miR-144-3p agomir on RCT and inflammation *in vivo*

**Figure 2:**
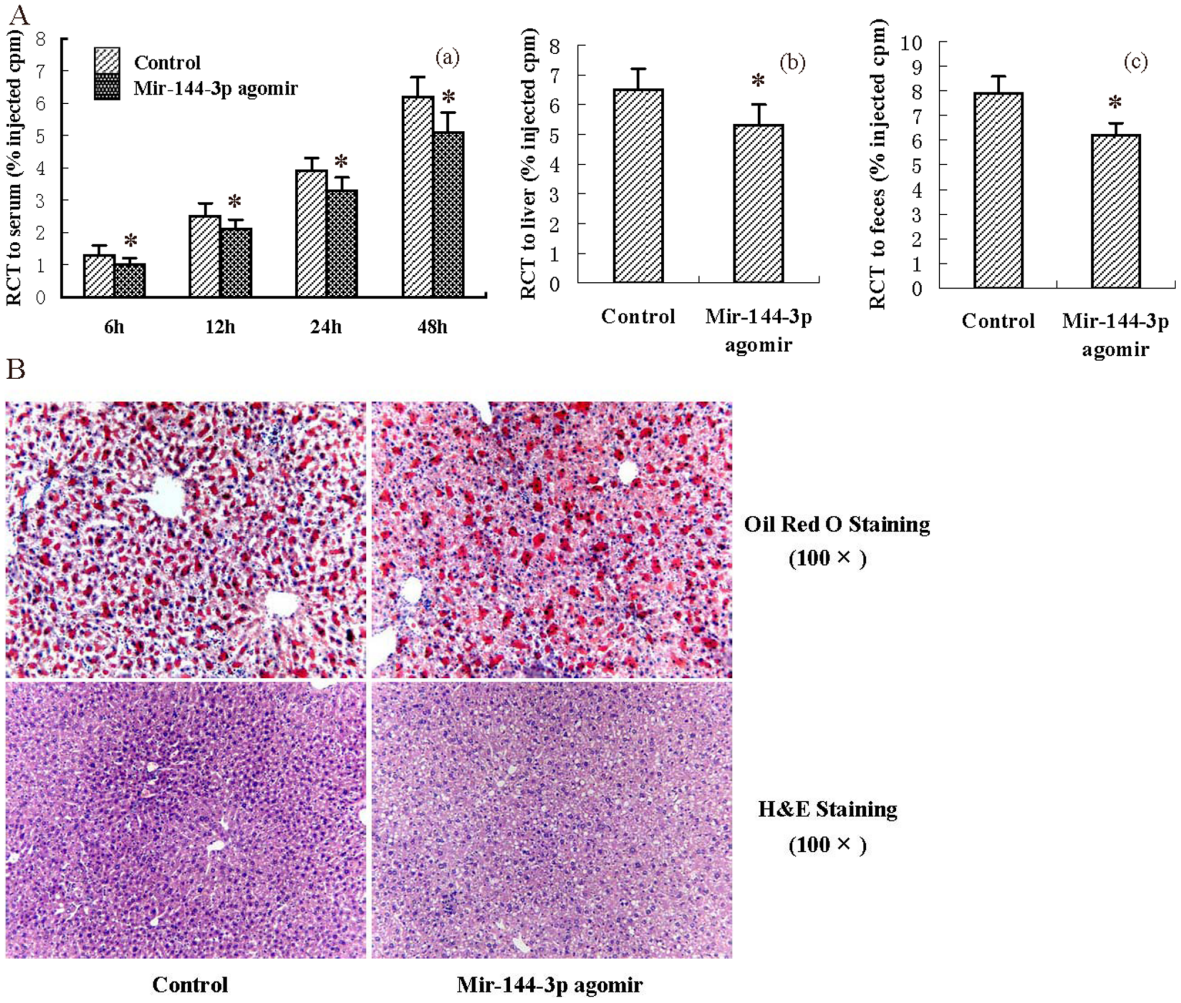
Effects of miR-144-3p on RCT and hepatic lipid deposition. (A) After 12 weeks of the indicated treatment, apoE^−/−^ mice were injected subcutaneously with ^3^H-cholesterol-labeled, Ac-LDL-loaded bone marrow-derived macrophages. Data are expressed as a percentage of the ^3^H-cholesterol tracer relative to that of total cpm tracer injected ± S.D.; n = 5. * *p*<0.05 vs. control group. (a) Time-course of ^3^H-cholesterol distribution in plasma. (b) Hepatic ^3^H-cholesterol tracer levels after 48 h. (c) Fecal ^3^H-cholesterol tracer levels. Feces were collected continuously from 0 to 48 h post-injection. (B) Liver cryosections were stained with Oil Red O and hematoxylin. Data are presented as mean values ± S.D.; n = 10. * *p*<0.05 vs. control group.

A combination of genetic and dietary manipulation results in extensive atherosclerotic disease in mice, which has many features in common with human lesions [19]. Since elevated levels of serum cholesterol are probably unique in being sufficient to drive the development of atherosclerosis in humans, we aimed to identify whether miR-144-3p agomir affects the lipid parameters in apoE^−/−^ mice administered HFD. As shown in Table 1, the levels of total cholesterol and HDL-C were markedly decreased in miR-144-3p agomir-treated mice, while changes in apoAI, apoB, LDL-C and VLDL-C concentrations were not statistically significant. Next, we explored the effects of miR-144-3p agomir on RCT efficiency in experimental mice. As shown, treatment of apoE^−/−^ mice fed a HFD with miR-144-3p agomir resulted in a dramatic decrease in RCT to serum, liver and feces. Interestingly, RCT to serum was downregulated by miR-144-3p agomir in a time-dependent manner (Fig. 2A). HDL mediates reverse transport of cholesterol to the liver for disposal. The liver is proposed to be a metabolic center for lipoproteins and cholesterol. Therefore, we subsequently analyzed the effects of miR-144-3p agomir on morphology and lipid content in the liver of apoE^−/−^ mice with hematoxylin and eosin (H&E) staining and Oil Red O staining, respectively. Representative images of randomly selected sections of liver stained for H&E and and Oil Red O in the control and miR-144-3p agomir groups are shown in Fig. 2B. Based on the number of vacuoles and nuclear size, increased lipid deposits in miR-144-3p agomir–treated animals were observed, compared with control animals. Oil Red O staining results additionally disclosed that the miR-144-3p agomir significantly accelerates hepatic lipid deposition in apoE^−/−^ mice. The effects of miR-144-3p agomir on the hepatic lipid content of apoE^−/−^ mice were further measured enzymatically. As shown in Table 2, both hepatic triglyceride and hepatic cholesterol levels in liver were increased in mice treated with miR-144-3p agomir, compared to control mice. We examined the inflammatory cytokine levels *in vivo* with a series of ELISA experiments. Treatment of apoE^−/−^ mice fed a HFD with the miR-144-3p agomir resulted in significant upregulation of plasma TNF-α, IL-1β and IL-6 by 54.3%, 45.6% and 68.4%, respectively (Table 3), consistent with *in vitro* findings. Our data collectively indicate that treatment with miR-144-3p mimics or agomir promotes pro-inflammatory cytokine production, both *in vivo* and *in vitro*.

**Table 1:** Effects of mir-144-3p agomir on plasma lipid and lipoprotein values in apoE^−/−^ mice.

	Control group (n = 10)	Mir-144-3p group (n = 10)
TG (mmol/L)	1.41±0.39	1.47±0.42
TC (mmol/L)	27.37±3.35	24.13±3.15*
HDL-C (mmol/L)	7.23±1.69	5.37±1.59*
LDL-C (mmol/L)	14.32±2.21	13.62±2.05
VLDL-C (mmol/L)	5.82±1.29	5.13±1.36
ApoA1 (g/L)	0.06±0.03	0.06±0.02
ApoB (g/L)	0.16±0.04	0.17±0.03

Data are expressed as mean ± S.D. The data were compared using the unpaired Student's *t*-test. * *p*<0.05 vs. control group.

**Table 2:** Effects of mir-144-3p agomir on hepatic lipid deposition in apoE^−/−^ mice.

	Control group (n = 10)	Mir-144-3p group (n = 10)
TC (mg/g tissue)	11.56±2.35	14.68±2.79*
TG (mg/g tissue)	19.43±2.32	20.57±2.16

Data are expressed as mean ± S.D. The data were compared using the unpaired Student's *t*-test. * *p*<0.05 vs. control group.

**Table 3:** Effects of mir-144-3p agomir on plasma cytokine levels in apoE^−/−^ mice.

	Control group (n = 10)	Mir-144-3p group (n = 10)
IL-1β (pg/mL)	17.63±3.98	25.71±6.69 *
IL-6 (pg/mL)	86.27±9.63	145.31±19.66*
TNF-α (pg/mL)	15.26±5.29	23.56±7.89*

Data are expressed as mean ± S.D. The data were compared using the unpaired Student's *t*-test. * *p*<0.05 vs. control group.

### 3. Effect of miR-144-3p agomir on pathological progress of atherosclerosis in apoE^−/−^ mice

**Figure 3:**
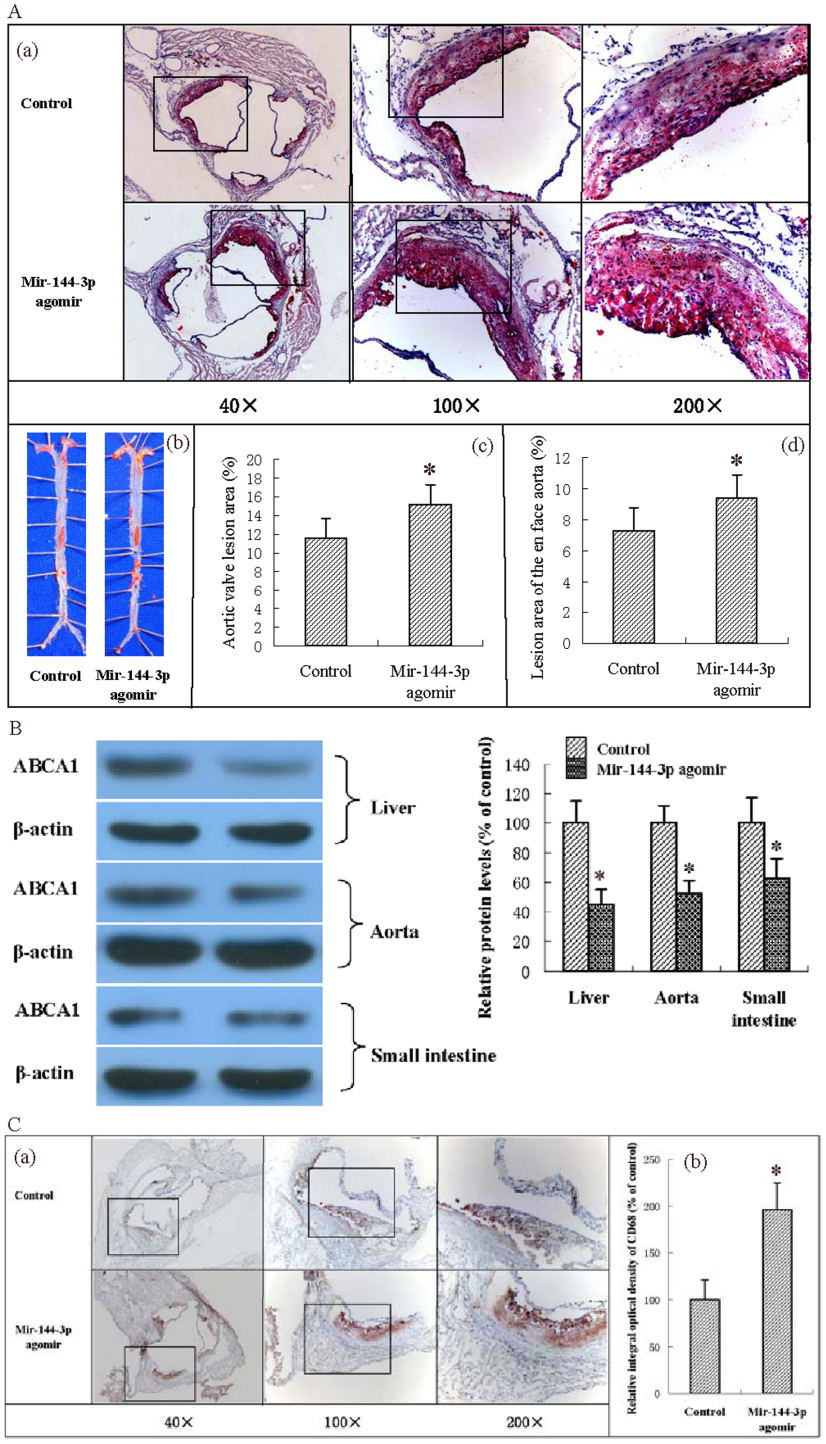
Effects of miR-144-3p on atherosclerosis initiation and development in apoE^−/−^ mice. (A) (a) Representative staining of aortic sinus with Oil Red O. (b) Representative staining of en face aorta with Oil Red O. (c) Lesions in aortic valves were analyzed in apoE^−/−^ mice. (d) En face lesions were analyzed in apoE^−/−^ mice. Data are presented as mean values ± S.D.; n = 10. * *p*<0.05 vs. control group. (B) Protein levels in tissues of apoE^−/−^ mice were analyzed using western blot analysis. Data are presented as mean values ± S.D.; n = 5. * *p*<0.05 vs. control group. (C) (a) Cryo-sections of aortic valves from apoE^−/−^ mice were immunohistochemically stained for the macrophage marker, CD68. (b) The integral optical density of CD68 in aortic valve cryo-sections from apoE^−/−^ mice was analyzed. Data are presented as mean values ± S.D.; n = 10. * *p*<0.05 vs. control group.

Atherosclerosis is a progressive disease, and clinical events are usually associated with rupture or erosion of the plaque [20]. Accordingly, we investigated the impact of miR-144-3p agomir on atheromatous plaque formation in apoE^−/−^ mice fed HFD. As shown in Fig. 3A, quantification of Oil Red O-stained aortic sinus sections revealed that treatment with miR-144-3p agomir resulted in a significant increase in lesion area (by 30.95%) in apoE^−/−^ mice, compared with control mice. To further establish the negative effects of miR-144-3p agomir on atherosclerosis, Oil Red O-stained lesions in en face preparations of aortas were quantified. Treatment of apoE^−/−^ mice with the miR-144-3p agomir led to a significant increase in lesion area (by 28.77%), compared with control mice. Since liver, small intestine and aorta are the most ABCA1-rich organs, we further explored the effect of miR-144-3p agomir on ABCA1 protein levels in apoE^−/−^ mice. Notably, miR-144-3p agomir downregulated ABCA1 protein expression in liver, aorta and small intestine (Fig. 3B). In addition, we performed immunohistochemical staining to visualize macrophages in atherosclerotic lesions (using an antibody against CD68). As shown, the CD68+ cell number in miR-144-3p agomir-treated mice was significantly increased, compared with that in control mice, indicating markedly higher macrophage infiltration in lesions of apoE^−/−^ mice. These findings clearly indicate a negative effect of miR-144-3p agomir in atherosclerotic lesions of apoE^−/−^ mice.

### 4. Circulating miR-144-3p is associated with acute myocardial infarction

In view of the finding that miR-144-3p is implicated in atherosclerosis, we investigated the serum miR-144-3p concentrations in 50 subjects. The clinical and biochemical characteristics of these subjects are presented in Table 4. Patients with AMI had significantly higher levels of creatine phosphokinase, creatine phosphokinase-MB fraction, lactate dehydrogenase, aspartate aminotransferase, and alanine aminotransferase. Table 5 shows the univariate associations between serum miR-144-3p concentration and concentrations of other biochemical characteristics. The serum miR-144-3p concentration was negatively correlated with serum HDL levels, and positively correlated with serum glucose concentrations in all subjects. In addition, serum miR-144-3p was positively correlated with serum creatine kinase (CK), creatine kinase-MB fraction (CK-MB), lactate dehydrogenase (LDH) and aspartate aminotransferase concentrations in subjects with AMI. We analyzed the expression levels of miR-144-3p in AMI patients after the onset of symptoms and healthy adults at the time-points of 4 h, 8 h, 12 h, 24 h, 48 h, 72 h and 1 week, respectively, as shown in Table 6. Patients with AMI exhibited a 2.16 (±0.12)-fold, 13.52 (±3.87)-fold and 4.38 (±1.53)-fold increase in serum miR-144-3p at 4, 8 and 12 h, respectively. The ROC curve of mir-144-3p revealed a moderate ability to distinguish between the AMI and the healthy control group at 4, 8 and 12 h, with AUC values of 0.81, 0.86, and 0.83, respectively.

**Table 4:** Clinical characteristics of patients.

Characteristics	AMI	Healthy outpatient	*p* Value
Age (years)	63.5±8.6	62±7.3	>0.05
Male/female (n/n)	15/10	17/8	>0.05
Current smoking, n (%)	6 (24%)	5 (20%)	>0.05
Diabetes mellitus, n (%)	3 (12%)	2 (8%)	>0.05
Hypertension, n (%)	12 (48%)	9 (36%)	>0.05
Hyperlipidaemia, n (%)	3 (12%)	1 (4%)	>0.05
Systolic blood pressure (mmHg)	135±22.3	123±19.6	>0.05
Diastolic blood pressure (mmHg)	85±15	73±14	>0.05
Glucose (mmol/L)	6.75±1.63	5.90±1.56	>0.05
Creatine phosphokinase (IU/L)	1236±453	98±11	0.0002
Creatine phosphokinase-MB fraction (IU/L)	86.7±36.5	12.5±1.3	0.0005
Lactate dehydrogenase (IU/L)	532±96.2	223±16.8	0.0312
C-reactive protein (mg/dL)	1.6±0.5	1.0±0.3	>0.05
Aspartate aminotransferase (IU/L)	132.6±41.3	26.7±6.5	0.0006
Alanine aminotransferase (IU/L)	36.5±7.5	23.7±6.8	0.0435
Total cholesterol (mmol/L)	4.23±0.8	4.06±0.7	>0.05
Total triglyceride (mmol/L)	1.52±1.1	1.45±0.8	>0.05
High-density lipoprotein (mmol/L)	1.02±0.22	1.25±0.39	>0.05
Low- density lipoprotein (mmol/L)	2.71±0.67	2.46±0.69	>0.05
ApoA-I (g/L)	1.23±0.12	1.46±0.19	>0.05
ApoB (g/L)	1.12±0.28	1.06±0.21	>0.05
Platelets (×10^9^/L)	236±35	186±28	>0.05
Creatinine (µmol/L)	96±58	68±32	>0.05
Blood urea nitrogen (mg/dL)	22.3±2.2	19.6±1.9	>0.05
Hemoglobin (g/dL)	12.6±0.7	13.5±0.8	>0.05
Hemoglobin A1c (%)	6.2±0.3	6.3±0.4	>0.05

AMI indicates acute myocardial infarction; *p*-value indicates comparison between patients with healthy adults. Data are expressed as mean ± S.D. The data were compared using the unpaired Student's *t*-test.

**Table 5:** Associations (Pearson correlation coefficients) of the serum mir-144-3p concentration with biochemical characteristics of subjects.

	mir-144-3p			
	Healthy adult		AMI	
	r	*p*	r	*p*
Glucose (mmol/L)	0.312	0.041	0.337	0.038
Creatine phosphokinase (IU/L)	0.457	0.065	0.675	0.016
Creatine phosphokinase-MB fraction (IU/L)	0.556	0.081	0.625	0.033
Lactate dehydrogenase (IU/L)	0.223	0.735	0.351	0.012
C-reactive protein (mg/dL)	0.095	0.359	0.087	0.515
Aspartate aminotransferase (IU/L)	0.331	0.069	0.416	0.046
Alanine aminotransferase (IU/L)	0.312	0.617	0.331	0.143
Total cholesterol (mmol/L)	0.219	0.446	0.167	0.548
Total triglyceride (mmol/L)	0.326	0.361	0.296	0.225
High-density lipoprotein (mmol/L)	−0.217	0.008	−0.316	0.009
Low-density lipoprotein (mmol/L)	0.176	0.326	0.218	0.239
ApoA-I (g/L)	−0.047	0.661	−0.068	0.669
ApoB (g/L)	0.376	0.098	0.415	0.121
Platelets (×10^9^/L)	0.213	0.663	0.562	0.356
Creatinine (µmol/L)	0.127	0.823	0.125	0.669
Blood urea nitrogen (mg/dL)	0.337	0.325	0.413	0.652
Hemoglobin (g/dL)	0.175	0.256	0.215	0.338
Hemoglobin A1c (%)	0.326	0.078	0.412	0.083

**Table 6:** mir-144-3p [levels] in human AMI patients and healthy adults.

	AMI (4 h)	AMI (8 h)	AMI (12 h)	AMI (24 h)	AMI (48 h)	AMI (72 h)	AMI (1 w)
Change fold (vs. Healthy adult)	2.16±0.12	13.52±3.87	4.38±1.53	1.22±0.15	1.17±0.12	0.98±0.10	1.15±0.13
*p*-value	0.015	0.001	0.008				
AUC	0.81	0.86	0.83				

Plasma samples were collected at 4 h, 8 h, 12 h, 24 h, 48 h, 72 h and 1 week after the onset of symptoms, and the data expressed as mean values ± S.D. Values indicated a fold change of mir-144-3p level vs. that in the control group. Corresponding *p*-values were calculated using the independent-samples *t*-test. Missing *p*-values represent non-significant miRNA level changes. AUC indicates the area under the receiver operating characteristic (ROC) curve for discrimination between AMI and control groups.

## Discussion

Atherosclerotic cardiovascular disease remains a major and expensive health burden. A strong independent inverse relationship of plasma HDL levels with atherosclerotic cardiovascular disease has been reported. Although HDL has cardioprotective activity via multiple mechanisms, one major activity is its role in RCT [1]–[2]. which is controlled by ATP-binding cassette transporters, particularly ABCA1[4]–[5]. The protein level and activity of ABCA1 are modulated via both transcriptional and posttranscriptional processes. Members of a class of miRNAs have been recently identified as potent posttranscriptional regulators of ABCA1 [7]–[9]. In the present study, we identified a putative miRNA, miR-144-3p, which targets 3′UTRs of human ABCA1 and causes post-transcriptional repression. Our results showed that a miR-144-3p agomir effectively accelerates atheromatous plaque formation in apoE^−/−^ mice by simultaneously impairing RCT and promoting pro-inflammatory cytokine production.

Studies on cultural cells and animal models have shown that treatment of THP-1 macrophage-derived foam cells with miR-144-3p mimics upregulates total cholesterol, free cholesterol and the cholesterol ester content in cells. Consistently, treatment of apoE^−/−^ mice with miR-144-3p agomir resulted in both impairment of RCT and dramatic decrease in HDL-C in our experiments. Epidemiological and clinical studies have provided considerable evidence that a low level of HDL-C is one of the most important risk factors of atherosclerotic cardiovascular disease (CVD), excess cholesterol in cells is cytotoxic, and accumulation of cholesterol in the artery wall initiates lesions [1], [2], [21]. HDL is a multifunctional and heterogeneous class of particles that plays an essential physiological role as a vehicle for delivery of peripheral cholesterol to the liver, namely RCT, which is widely believed to account for the inverse relationship between plasma HDL-C level and risk for CVD [22]–[23]. In addition, HDL exerts a protective effect by inhibiting lipoprotein oxidation, while oxidative modification of HDL impairs its atheroprotective properties and oxidatively modified LDL contributes significantly to intima injury, monocyte recruitment and foam cell formation, all of which are critical events in the initiation and progression of atherosclerosis [24]. The miR-144-3p mimic or agomir induced significant upregulation of different types of cholesterol at the cellular level and downregulated HDL-C in the body, indicating a critical role of miR-144-3p in the complex homeostatic network in cholesterol metabolism. HDL components remove cellular cholesterol via multiple mechanisms involving passive diffusion, interactions with SR-B1, and transport activities of ABCs, in particular, ABCA1 [23], [25]. ABCA1 is a cell membrane protein that mediates the transport of cholesterol, phospholipids and other metabolites from cells to lipid-depleted HDL apolipoproteins [26]. This protein is a major determinant of plasma HDL levels and a potent atheroprotective factor [27], [28]. In the present study, treatment of THP-1 macrophages with miR-144-3p mimics resulted in a marked decrease in cholesterol efflux to both apoAI and HDL. In addition, miR-144-3p agomir suppressed ABCA1 protein expression in liver, aorta and small intestine in apoE^−/−^ mice fed a HFD, and induced a dramatic decrease in RCT to serum, liver and feces. ABCA1 is highly expressed in the liver and tissue macrophages. Liver ABCA1 initiates the formation of HDL particles, while arterial macrophage ABCA1 mediates reverse transport cholesterol and intestinal ABCA1 functions to generate HDL particles that transport dietary cholesterol to the liver. Consequently, decreased ABCA1 expression and activity in organs or tissues would negatively affect the diverse atheroprotective functions of the lipoprotein subclass [29], [30]. MiR-144-3p may bind to partially complementary sequences in the 3′UTRs of human ABCA1 and downregulate expression. Moreover, the relative efficiency of the RCT pathway is affected by miR-144-3p mimics or agomir treatment. Although the mechanistic details remain to be defined, this miRNA is proposed to play a critical role in the regulation of cholesterol homeostasis and anti-atherosclerosis.

Accumulating experimental and clinical evidence supports the theory that atherosclerosis is a form of chronic inflammation, and macrophages play a key role. It is widely accepted that recruitment of macrophages and their subsequent foam cells are the major cellular events contributing to the pathological process of atherosclerosis [3]. Recent research has indicated that ABCA1, in addition to lipid transport activity, not only influences inflammatory signaling pathways indirectly by modifying cell surface lipid domains, but also directly acts as an anti-inflammatory receptor by inducing signaling through the Janus kinase 2(JAK2)/STAT3 pathway in response to binding lipid poor apoAI [31], [32]. Furthermore, receptors other than ABCA1 that modulate HDL levels and flux of cholesterol through the RCT pathway are reported to have anti-inflammatory properties [33], [34]. As a result of miR-144-3p mimic or agomir treatment, expression levels of ABCA1 in cultured cells or experimental mice were remarkably inhibited in our study. Treatment of THP-1 macrophage-derived foam cells with miR-144-3p mimics increased inflammatory cytokine levels, including those of TNF-α, IL-1β and IL-6. Similarly, the plasma concentrations of TNF-α, IL-1β and IL-6 in apoE^−/−^ mice fed HFD were dramatically enhanced upon treatment with miR-144-3p agomir. An insight into the pathogenesis of atherosclerosis revealed that uptake of highly oxidized LDL particles by scavenger receptors in macrophages leads to foam cell formation, and expression of scavenger receptors is regulated by cytokines, such as TNF-α and interferon-γ (IFN-γ) [35]. Alternatively TNF-α increases the risk of CAD by interfering with the thrombotic process owing to enhancement of procoagulant activity, and suppresses the antithrombotic protein C pathway in endothelial cells [36]. IL-1β induces the production of a broad spectrum of cytokines and chemokines, as well as expression of adhesion molecules on endothelial cells, leading to the recruitment of inflammatory cells [37]. Circulating levels of IL-6 represent a strong independent marker of increased mortality among patients with unstable coronary artery disease, and may be useful in directing subsequent care [38]. The miR-144-3p mimic/agomir induced a dramatic increase in the relative levels of types of inflammatory factors levels, both *in vitro* and *in vivo*, confirming its pro-inflammatory property, and therefore, activity as a potent negative regulator of CVD. In addition, treatment of apoE^−/−^ mice with miR-144-3p agomir resulted in increased macrophage infiltration in lesions. As discussed previously, macrophages may initially function as a protective factor, but present a burden to the arterial wall owing to excessive accumulation of ox-LDL. Here, we reported that the miR-144-3p agomir dramatically accelerates the progress of atheroma in apoE^−/−^ mice by simultaneously increasing the CD68+ cell content in plaque areas and promoting pro-inflammatory cytokine production in plasma.

As plaque rupture and thrombosis convert chronic inflammatory conditions to an acute clinical event and diagnosis with optimal rapidity and sensitivity will benefit both patients and doctors, blood protein markers have been included in the diagnosis or assessment of AMI, including CK-MB, troponin T (TnT) and troponin I (TnI) [39], [40]. A clinical investigation by our group revealed that the circulating miR-144-3p concentration is positively correlated with serum CK, CK-MB, LDH and aspartate aminotransferase in AMI subjects. Additionally, the serum miR-144-3p level was elevated within 4 h, reached a peak at 8 h, and was suppressed within 12 h after the onset of chest pain. The ability of miR-144-3p to distinguish between the AMI and control groups was observed from the ROC curves, with AUC values of 0.81, 0.86 and 0.83, respectively. Thus, the plasma concentrations of miR-144-3p showed a significant correlation with those of CK, CK-MB and LDH, a series of classic markers of myocardial injury. Although these markers are robust diagnostic and prognostic indicators in the setting of suspected AMI, there are still perceived limitations [39], [41]. Except for rapidity, the ideal nucleotide blood biomarker of AMI should be cardiomyocyte-specific, detectable, and stable in blood. Serum miR-144-3p investigated in the present study corresponds to the above requirements. It is important to note that the current investigation involved a relatively small sample size, and further experimental studies are therefore necessary to explore the underlying mechanisms. Moreover, serum mir-144-3p was negatively correlated with serum HDL and positively correlated with serum glucose concentrations in all subjects. The cardioprotective role of HDL is widely accepted, while high serum glucose is one of the major risk factors of atherosclerosis, and most patients with diabetes die from complications of atherosclerosis [42]. Thus, whether miR-144-3p may contribute to increased risk of cardiovascular disease in diabetics and the underlying mechanism should to be further investigated. Circulating miRNAs are emerging as novel biomarkers for AMI [43]–[45]. Huang *et al* showed that lower levels of miR-320b and miR-125b were associated with increased occurrence of AMI [46]. Xiao *et al* demonstrated that serum miR-208a and miR-499 were elevated after AMI and might be potential biomarkers for AMI [47]. Long *et al* found that circulating miR-30a and miR-195 were highly expressed while let-7b was significantly lower in AMI patients at 8 h after onset of AMI and suggested that the plasma concentration of miR-30a, miR-195 and let-7b could be potential indicators for AMI [48]. In the present study, we found that serum miR-144-3p levels were markedly increased in AMI patients after the onset of symptoms and revealed a positive correlation of circulating miR-144-3p with serum CK, CK-MB, LDH and AST in subjects with AMI. These results collectively imply that miR-144-3p plays critical roles in the pathophysiological processes of AMI, and circulating miR-144-3p may serve as a promising biomarker for AMI diagnosis.

## Conclusions

Atherosclerosis is not simply an inevitable degenerative consequence of aging, but rather a chronic inflammatory condition. Mir-144-3p targeting ABCA1, the doorkeeper of RCT, resulted in impaired cholesterol efflux and boosted inflammatory response, which effectively accelerated the occurrence and progression of atherosclerosis in apoE^−/−^ mice fed HFD. In conclusion, our study supports the significance of miR-144-3p as a promising therapeutic target for atherosclerosis and potential candidate biomarker of AMI.
